# Mutations in the *ARAP3* Gene in Three Families with Primary Lymphedema Negative for Mutations in Known Lymphedema-Associated Genes

**DOI:** 10.1155/2020/3781791

**Published:** 2020-08-25

**Authors:** Maurizio Ricci, Rita Compagna, Bruno Amato, Sercan Kenanoglu, Dominika Veselenyiova, Danjela Kurti, Mirko Baglivo, Syed Hussain Basha, Roberta Serrani, Giacinto Abele Donato Miggiano, Barbara Aquilanti, Giuseppina Matera, Giuseppe Marceddu, Valeria Velluti, Lucilla Gagliardi, Munis Dundar, Juraj Krajcovic, Matteo Bertelli

**Affiliations:** ^1^Division of Rehabilitation Medicine, Azienda Ospedaliero-Universitaria, Ospedali Riuniti di Ancona, Italy; ^2^Department of Public Health, University of Naples Federico II, Naples, Italy; ^3^Department of General and Geriatric Surgery, University of Naples “Federico II”, Naples, Italy; ^4^Department of Medical Genetics, Faculty of Medicine, Erciyes University, Kayseri, Turkey; ^5^MAGI Euregio, Bolzano, Italy; ^6^Department of Biology, Faculty of Natural Sciences, University of Ss. Cyril and Methodius in Trnava, Trnava, Slovakia; ^7^MAGI-Balkan, Tirana, Albania; ^8^Innovative Informatica Technologies, Hyderabad, India; ^9^UOC Nutrizione Clinica, Fondazione Policlinico Universitario A. Gemelli IRCCS, Rome, Italy; ^10^Centro di Ricerche in Nutrizione Umana, Università Cattolica Sacro Cuore, Rome, Italy; ^11^EBTNA-Lab, Rovereto, Italy; ^12^MAGI's Lab, Rovereto, Italy

## Abstract

**Background:**

ARAP3 is a small GTPase-activating protein regulator, which has important functions in lymphatic vessel organogenesis and modulation of cell adhesion and migration. Mutations in the *ARAP3* gene are associated with impaired lymphatic vessel formation.

**Objective:**

The aim of our study was to determine the genotypes of lymphedema patients in relation to variants in the *ARAP3* gene in order to explore its role in the development of lymphedema.

**Methods and Results:**

We applied next-generation sequencing to DNA samples of a cohort of 246 Italian patients with lymphatic malformations. When we tested probands for known lymphedema genes, 235 out of 246 were negative. Retrospectively, we tested the DNA of these 235 patients for new candidate lymphedema-associated genes, including *ARAP3*. Three out of 235 probands proved to carry rare missense heterozygous variants in *ARAP3*. In the case of two families, other family members were also tested and proved negative for the *ARAP3* variant, besides being unaffected by lymphedema. According to *in silico* analysis, alterations due to these variants have a significant impact on the overall structure and stability of the resulting proteins.

**Conclusions:**

Based on our results, we propose that variants in *ARAP3* could be included in genetic testing for lymphedema.

## 1. Introduction

ARAP3, also known as CENTD3 or DRAG1, is a GTPase-activating protein (GAP) encoded by the *ARAP3* gene, which consists of 35 exons and maps to 5q31.3. ARAP3 has a multidomain structure, containing ArfGAP and RhoGAP domains, ankyrin repeats, and the pleckstrin homology domain 3 (PH3), and is therefore capable of phosphoinositide binding [1]. While the ArfGAP and RhoGAP domains work together to shape the cell cytoskeleton, PH3 facilitates cell signaling by binding to specific phosphoinositides [2].

ARAP3 was first identified for its capacity to bind phosphatidylinositol (3,4,5)-triphosphate (PIP3) in porcine leukocytes [3]. Its activity is controlled by PI3K (phosphoinositide 3-OH kinase), which regulates its catalytic activity and its localization in the cell, and by RAP-GTP, which controls its RhoGAP activity [3]. After activation by PI3K, ARAP3 undergoes translocation to the cell membrane, where its substrates RhoA-GTP and ARF6-GTP are located. ARAP3 depletion prevents its substrate GTPases from activating. In endothelial cells, this leads to changes in cell shape [4, 5].

The expression and function of *ARAP3* have been studied in mice and zebrafish models [6] (Table 1). ARAP3 is irreplaceable in lymphatic vascular development, where it acts as a regulator of lymphatic vessel organogenesis and also modulates cell adhesion and migration. During embryo development, lymphatic vessels are formed by lymphangiogenesis from preexisting veins. Several molecular factors are crucial for the differentiation of endothelial cells into lymphatic endothelial cells (LECs) [7, 8]. The best known of these factors is VEGFR3 (vascular endothelial growth factor 3), which is important for the sprouting of precursor LECs that later become adult LECs [9]. ARAP3 seems to act downstream of VEGFR3 during lymphangiogenesis [6]. Depletion of ARAP3 in zebrafish leads to fewer precursor LECs sprouting from the cardinal vein, resulting in impaired lymphatic vessel formation. Despite the close link between lymphatic and blood vessel development, ARAP3-deficient zebrafish shows a normal blood vascular phenotype.

However, *ARAP3*-null mice show impaired blood vessel development as well as lymphatic system malformations and die prematurely [10]. *ARAP3* dysregulation has also been reported in a mouse model of lymphatic diseases [6]. These findings provide evidence that ARAP3 is necessary for normal lymphangiogenesis during embryo development in mouse and zebrafish models.

The *ARAP3* gene has not yet been associated with any pathological phenotype in OMIM, although its function is implicated in the lymphatic system. Several genome-wide association studies (GWAS) have been done to associate *ARAP3* polymorphisms with different traits. Cerhan et al. investigated SNPs in multiple genes in the context of B-cell lymphoma. They performed a comprehensive meta-analysis and reported a single nucleotide *ARAP3* variant rs79464052 associated with susceptibility for diffuse large B-cell lymphoma [11]. This type of lymphoma affects the lymphatic system and is characterized by fast growing lymph nodes deep in the body or in the peripheral lymph nodes. *ARAP3* polymorphism is suspected to indicate susceptibility to this disease.

Lymphedema is a lymphatic system disorder that manifests as edema, usually affecting the extremities. Other symptoms involve inflammation, fat accumulation, and fibrosis [12]. Lymphedema is a progressive disease that develops due to impaired fluid flow caused by lymphatic system malformations. Lymphedema patients typically show excessive lymphangiogenesis, lymphatic vessel defects, and lymph node hypoplasia/hyperplasia [13].

The available data provides evidence that the *ARAP3* gene is necessary for normal lymphatic system development. Although the details of its function are still uncertain and more studies are needed, the importance of its role in lymphangiogenesis is apparent.

We tested 235 Italian lymphedema patients, who had previously proved to be negative for variants in known lymphedema genes, for variants in *ARAP3* [14]. We studied the genotype-phenotype relationship in patients carrying variants in *ARAP3* in order to investigate whether *ARAP3* could qualify as a candidate gene for lymphedema.

## 2. Materials and Methods

### 2.1. Clinical Evaluation

We retrospectively analyzed samples from 246 Caucasian patients diagnosed with primary lymphedema in hospitals across Italy. No consanguinity was reported in their families. Clinical diagnosis of lymphedema was according to generally approved clinical criteria. Genetic testing was performed on germline DNA extracted from the saliva or blood of probands.

### 2.2. Genetic Analysis

A custom-made oligonucleotide probe library was designed to capture all coding exons and flanking exon/intron boundaries (±15 bp) of 29 genes known to be associated with lymphedema. We added the candidate gene *ARAP3* to our panel. Variants with likely clinical significance identified in DNA of probands were confirmed by bidirectional Sanger sequencing on a CEQ8800 Sequencer (Beckman Coulter).

We searched the international databases dbSNP (http://www.ncbi.nlm.nih.gov/SNP/) and Human Gene Mutation Database professional (HGMD; http://www.biobase-international.com/product/hgmd) for all nucleotide changes. *In silico* evaluation of the pathogenicity of nucleotide changes in exons was performed using the Variant Effect Predictor tool (http://www.ensembl.org/Tools/VEP) and Varsome (http://www.varsome.com). Minor allele frequencies (MAF) were checked in the Genome Aggregation Database (gnomAD) (http://gnomad.broadinstitute.org/). All variants were evaluated according to the American College of Medical Genetics and Genomics guidelines [15]. Detailed pretest genetic counseling was provided to all subjects, who were then invited to sign specific informed consent to use their anonymized genetic results for research.

### 2.3. *In Silico* Analysis

**Figure 1:**
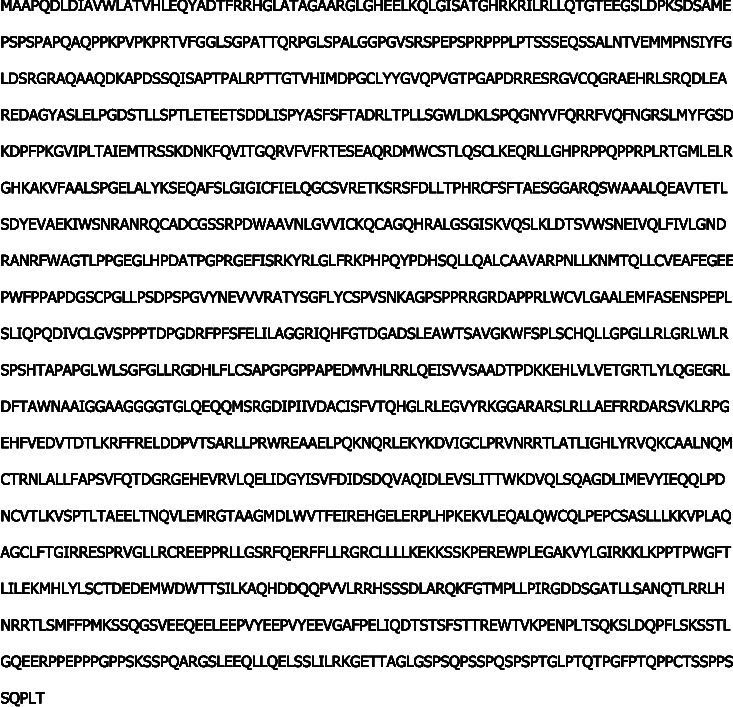
Primary amino acid sequence used to search for templates and build models.

The primary amino acid sequence of ARAP3 in FASTA format (Figure 1) was used to search the Swiss-Model template library (SMTL) version 2019-10-24 and Protein Data Bank (PDB) released 2019-10-18 [16] with BLAST (Basic Local Alignment Search Tool) [17] and HHBlits [18] for evolution-related structures matching the given ARAP3 sequence. Models based on target-template alignment were built using ProMod3 of the Swiss-Model server [19]. Coordinates conserved between target and template were copied from the template to the model. Insertions and deletions were remodeled using a fragment library. Side chains were then rebuilt. Finally, the geometry of the resulting model was regularized using the CHARMM27 force field [20]. If loop modeling with ProMod3 failed, an alternative model was built with PROMOD-II [21]. Global and per-residue model quality was assessed using the QMEAN scoring function [22]. The BioVia Discovery Studio Visualizer v17.2 [23] was used to visualize the modeled protein, to mutate the targeted amino acids, and to analyze molecular level interactions.

## 3. Results

### 3.1. Clinical and Genetic Evaluation

In this study, we analyzed 235 patients for variants in the *ARAP3* gene. These patients, diagnosed with lymphedema, were negative to genetic testing for variants in known lymphedema genes [14]. Three different heterozygous variants in *ARAP3* were found in three probands. All cases were sporadic with no family history of lymphedema. In families 1 and 2, family members were also tested, but with a negative result. The *ARAP3* variants we identified therefore do not segregate in the families. The clinical features of the probands are summarized in Table 2.

**Figure 2:**
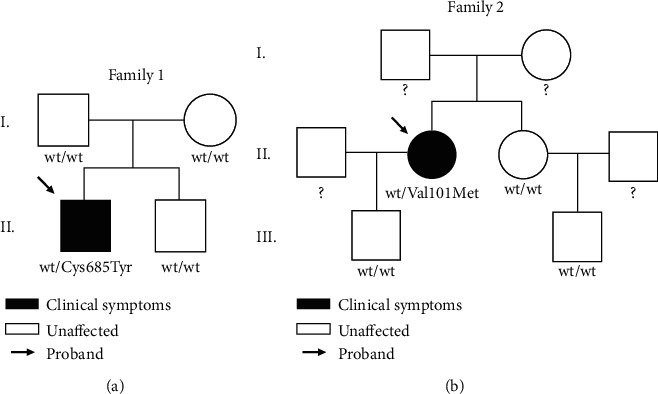
Pedigrees of families with *ARAP3* variants. (a) Family 1: proband affected with lymphedema carries an *ARAP3* variant; three other unaffected family members do not carry the variant. (b) Family 2: proband diagnosed with lymphedema and an *ARAP3* variant. Three other unaffected family members tested negative.

The first proband is a male diagnosed with primary lymphedema at 25 years of age. The patient undergoes a clinical examination after an episode of lymphangitis in the left limb accompanied by fever. One week after the resolution of this episode, edema occurred in the left lower limb and scrotum. The proband carries a missense variant NP_071926.4:p.Cys685Tyr, not identified in dbSNP and with unknown allele frequency. Three unaffected family members were also tested, and they were all negative for the variant (Figure 2(a)), suggesting a *de novo* mutation.

The second proband is a female with left lower limb lymphedema, diagnosed at 39 years. The patient noticed heaviness and swelling of the left lower limb over the last 2 months. The left lower limb is swollen and shows the presence of pitting edema extending from the thigh down to the foot. She carries a missense variant NP_071926.4:p.Val101Met. This single nucleotide variant is known in dbSNP (rs200702800), and gnomAD lists its frequency as 0.00004. Three unaffected family members were tested and do not carry the variant (Figure 2(b)).

The third proband is a male with right lower limb and foot edema diagnosed at 49 years. The patient complains of progressive swelling of his right leg over the last 6 months. An episode of lymphangitis has been successfully cured. A missense single nucleotide variant was identified: NP_071926.4:p.Arg1478Gln, listed in dbSNP as rs147992246 with a reported frequency of 0.00202.

**Figure 3:**
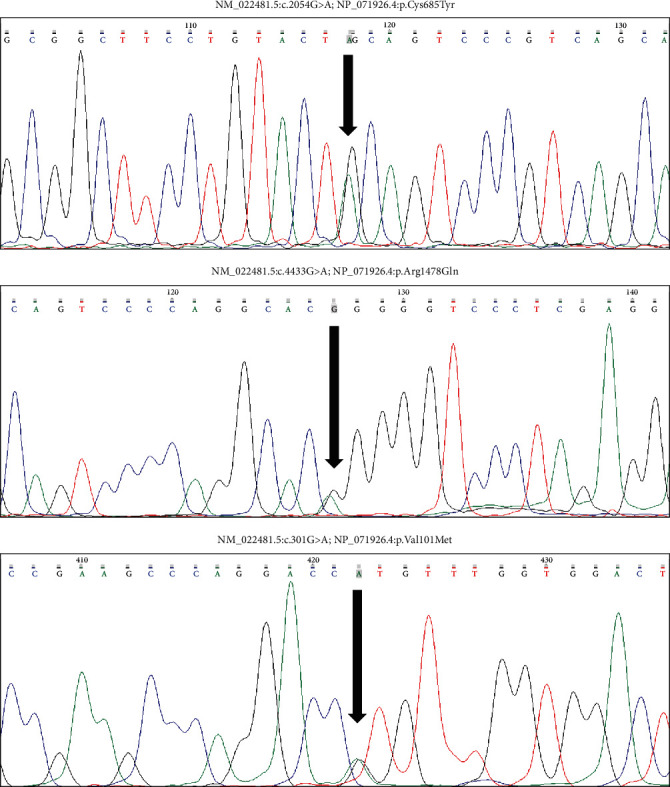
Sanger sequences of three identified variants in *ARAP3*.

**Figure 4:**
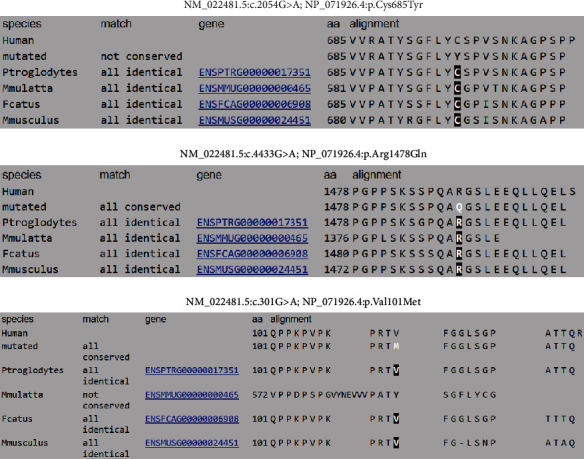
Conservative analysis of three identified variants in *ARAP3*. This residue is preserved in the most relevant mammals including primates, felines, and model rodents such as *Mus musculus*.

All the variants were confirmed by Sanger sequencing (Figure 3). Conservative analysis shows that the residues affected by the variants are preserved in the main mammalian models (Figure 4).

### 3.2. Template Selection and Model Building

Template search with BLAST and HHBlits was performed against the Swiss-Model template library (SMTL, last update: 2019-10-24, last included PDB release: 2019-10-18). The target sequence was sought with BLAST against the primary amino acid sequence (Figure 1) in the SMTL. A total of 697 templates were found that matched with various sequence identity and quality percentages. Details of the top 10 templates are shown in Table 3.

### 3.3. *In Silico* Analysis

*In silico* analysis showed that the *ARAP3* gene coded structure with Cys685 showed significant differences in interactions with Tyr685. This amino acid change causes a gain in interaction strength. While Cys685 formed two interactions with Ile682 and Arg690, Tyr685 shows five interactions: two direct hydrogen bonds with Trp662, Ile682, and Arg690 along with pi interactions with Leu658 and Ile682.

**Figure 5:**
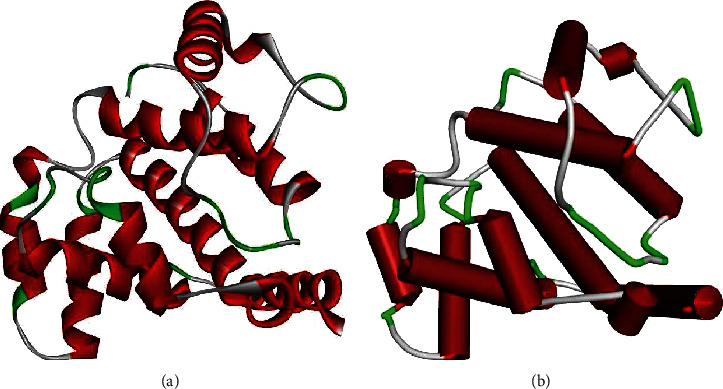
Modeled structure of *ARAP3* (ArfGAP with RhoGAP domain, ankyrin repeat, and PH domain 3) represented in (a) ribbon and (b) schematic. Green regions represent beta sheets, white regions represent loops, and red regions represent alpha helices.

**Figure 6:**
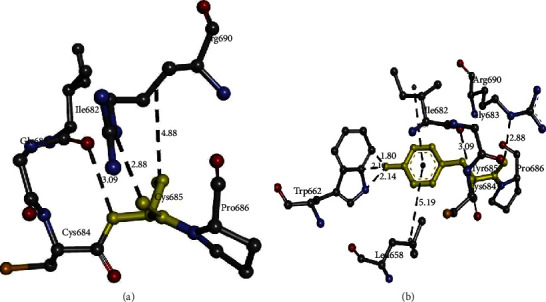
Molecular interactions of (a) Cys685 and (b) Tyr685 (highlighted in yellow) of the modeled ARAP3 protein with adjacent interacting residues.

Based on the percentage of sequence identity, similarity, and best quality square, the 5jd0.1.A chain was selected to align the template and query sequences for model building. The model is shown in Figure 5. We then used the Discovery Studio Visualizer to generate the Cys685Tyr mutated version of the modeled structure. For lack of a template, the Arg1478Gln region was not modeled. The modeling of Val101Met was not possible due to limitation in the template region. Molecular level interactions between native/mutated residues and interacting residues are shown as snapshots in Figure 6. Details of the residues involved in interactions, the type of bonds they formed, and bond lengths in angstrom units are shown in Table 4.

## 4. Discussion and Conclusions

Lymphedema is a chronic disease caused by the accumulation of protein-rich interstitial fluid due to lymphatic system insufficiency associated with swelling, weight gain, tightness, and pain in the extremities [24]. It is a difficult condition to treat. Early diagnosis and treatment are important for disease management and to prevent complications. Correct diagnosis is crucial: identifying candidate genes can help patients and determine predisposition for lymphatic system malformations in the healthy population and family members of lymphedema patients.

In the study, we genotyped 246 Caucasian patients for variants in known genes associated with lymphedema; 235 tested negative, and their samples were further tested for variants in candidate genes including *ARAP3*.

We found three probands (3/235; 1.28%) with three different heterozygous missense single nucleotide variants in *ARAP3*. In family 1, the parents and a brother of the proband were also tested, but none of them carried the variant, suggesting that the variant arose *de novo*. In family 2, we tested the son, sister, and nephew of the proband. The variant was not identified in these family members; the parents of the proband were not tested. No family members of the third proband are available for analysis.

*In silico analysis* allows us to speculate that overall protein conformation is somehow altered by differences in interactions with nearby residues, leading to functional defects in the protein.

To the best of our knowledge, *ARAP3* has never previously been implicated in the development of lymphedema in humans. Known associations between *ARAP3* and the human phenotype are limited, coming only from GWAS focused on traits like birth weight, carcinogenesis, and neural development [11, 25, 26]. These studies have been inconclusive regarding *ARAP3* function in these processes.

Studies on animal models have been more fruitful. As demonstrated in a mouse model of lymphatic diseases (HLT model: hypotrichosis-lymphedema-telangiectasia), *ARAP3* is dysregulated in these disorders. This suggests that the normal function of *ARAP3* is important in preventing lymphatic diseases. More evidence has been found in zebrafish, where *ARAP3* is necessary for normal development of lymphatic vessels. Loss of *ARAP3* function leads to a restricted number of LEC precursors that give rise to mature LECs. These findings strongly implicate *ARAP3* in the correct development of the lymphatic system during embryogenesis. The work on animal models done by Kartopawiro et al. [6] is to our knowledge the only study directly connecting ARAP3 function with the anomalies of lymphatic system, so far. This shows how limited the information is about the role of ARAP3 in the development of lymphatics in experimental models as well as in humans. Our work implies that ARAP3 variants discussed here can cause anomalies of the lymphatics, and therefore, this work provides new evidence for the involvement of the *ARAP3* gene in lymphatic system development.

This review of the literature and our results lead us to propose that *ARAP3* may be important in normal lymphatic vascular formation in humans and can therefore determine a predisposition for lymphedema caused by lymphatic system malformations. We suggest including *ARAP3* in the genetic testing for patients affected by lymphedema negative for known genes associated to the disease to confirm our results.

## Figures and Tables

**Table 1:** Phenotype associated with the *Arap3* gene in different organisms.

Organism	Gene	Function	Lethality	Lymphatic phenotype
Mouse	*Arap3*	Protein coding	Conditional *Arap3* knockout lethal during gestation, impaired vascular development [10]	Lymphatic anomalies, dysregulation of *Arap3* in a mouse model of lymphatic disease [6]
Zebrafish	*arap3*	Protein coding	/	*arap3* fewer precursor lymphatic endothelial cells sprout from the cardinal vein [6]

**Table 2:** Clinical features of the probands.

Sex	Age	Clinical features	Age of onset	Family history	Computational prediction	Variant nomenclature
M	32	Left lower limb and scrotal lymphedema	25	No	Pathogenic predictions from FATHMM-MKL, MutationAssessor, and MutationTaster	NM_022481.5:c.2054G>A NP_071926.4:p.Cys685Tyr
F	58	Left lower limb lymphedema	39	No	Pathogenic predictions from DANN and SIFT	NM_022481.5:c.301G>A NP_071926.4:p.Val101Met
M	59	Right lower limb edema, foot edema	49	No	No pathogenic predictions	NM_022481.5:c.4433G>A NP_071926.4:p.Arg1478Gln

**Table 3:** Top ten models for 3D modeling of the ARAP3 structure.

Template	Seq identity	Oligo-state	QSQE	Found by	Method	Resolution	Seq similarity	Coverage	Description
3lju.1.A	22.05	Monomer	—	HHblits	X-ray	1.70 Å	0.30	0.21	ArfGAP with dual PH-domain-containing protein 1
3mdb.2.B	21.82	Monomer	—	HHblits	X-ray	2.95 Å	0.30	0.21	ArfGAP with dual PH-domain-containing protein 1
3feh.1.A	22.05	Monomer	—	HHblits	X-ray	1.90 Å	0.30	0.21	Centaurin-alpha-1
5jd0.1.A	100.00	Monomer	—	BLAST	X-ray	2.30 Å	0.60	0.13	ArfGAP with RhoGAP domain, ANK repeat, and PH-domain-containing protein 3
3mdb.1.B	21.82	Monomer	—	HHblits	X-ray	2.95 Å	0.30	0.21	ArfGAP with dual PH-domain-containing protein 1
5jcp.1.A	100.00	Monomer	—	BLAST	X-ray	2.10 Å	0.60	0.13	ArfGAP with RhoGAP domain, ANK repeat, and PH-domain-containing protein 3, linker, transforming protein RhoA
5jd0.1.A	100.00	Monomer	—	HHblits	X-ray	2.30 Å	0.60	0.13	ArfGAP with RhoGAP domain, ANK repeat, and PH-domain-containing protein 3
5jcp.1.A	100.00	Monomer	—	HHblits	X-ray	2.10 Å	0.60	0.13	ArfGAP with RhoGAP domain, ANK repeat, and PH-domain-containing protein 3, linker, transforming protein RhoA
5c5s.1.A	27.41	Monomer	—	HHblits	X-ray	2.20 Å	0.34	0.13	Unconventional myosin-IXb
5c5s.2.A	27.41	Monomer	—	HHblits	X-ray	2.20 Å	0.34	0.13	Unconventional myosin-IXb

**Table 4:** Details of molecular interactions of Cys685 and Tyr685 of the modeled ARAP3 protein with adjacent interacting residues.

S. no.	Mutation	Amino acid	Molecular interactions observed	Bond length in angstroms	Bond type
1	Cys685Tyr	Cys685	Cys685:N-Ile682:O	3.09	H-bond
2			Arg690:N-Cys685:O	2.88	H-bond
3			Cys685-Arg690	4.88	Hydrophobic
4		Tyr685	Trp662:N-Tyr685:O	2.14	H-bond
5			Trp662:C-Tyr685:O	2.10	H-bond
6			Trp662:C-Tyr685:O	1.80	H-bond
7			Tyr685:N-Ile682:O	3.09	H-bond
8			Arg690:N-Tyr685:O	2.88	H-bond
9			Tyr685-Leu658	5.19	Pi interaction
10			Tyr685-Ile682	5.37	Pi interaction

## Data Availability

The data used to support the findings of this study are included within the article.
